#  Frequency and trends of hospital treated pesticide poisonings in Germany 2000–2014

**DOI:** 10.3205/000254

**Published:** 2017-08-14

**Authors:** Susanne Moebus, Wolfgang Boedeker

**Affiliations:** 1 Centre for Urban Epidemiology, University Clinic, University of Duisburg-Essen, Essen, Germany; 2 EPICURUS – Impact Assessment, Essen, Germany

**Keywords:** pesticides, poisoning, mortality, morbidity, case fatality, hospital discharge

## Abstract

**Objectives:** To analyze the occurrence, trends, and patterns of hospital treated pesticide poisonings in Germany

**Methods:** Data from the German diagnoses statistics of hospital discharges are analyzed for the years 2000–2014. ICD code specific numbers of cases as well as rates per one million and case-fatality ratios are calculated. The number of fatal pesticides poisonings is compared to the official German causes-of-death statistics.

**Results:** During 2000 and 2014 overall 2,871 pesticides poisonings were treated in hospitals with 191 cases per year on average. The rate per 1 million dropped from 2.74 in 2000 to 1.38 in 2014. On average 5% of pesticides poisonings were fatal, this percentage also approximately halved from 2000 to 2014. The majority of pesticide poisonings occurred in men. In both sexes about 70% of all pesticide poisonings occurred below 55 years and one third of patients were younger than 25 years. With respect to fatal poisonings men shared almost 80% of incidents and more than 70% of cases occurred above 55 years of age. There is poor agreement between the different data sources studied. On average only 24% of the fatal pesticide poisoning in the mortality statistics were seen in the diagnoses statistics of hospital discharges.

**Conclusions:** This study shows that the decrease of pesticide poisonings in Germany applies to fatal and non-fatal incidents. There seems to be a decrease also in the case fatality ratio which might point to positive preventive effects by reducing the availability of toxic pesticides. Fatal pesticides poisonings prevails in elder men while non-fatal pesticide poisoning more often affects the younger population. These different patterns should be addressed when improving preventive strategies. The discrepancy between the different data bases with respect to fatal poisoning might be explained by intoxications not admitted to a hospital. However, the difference seems rather high and calls for a deeper investigation. The ICD version 10 does not provide codes allowing for pesticide-specific analyzes of poisonings. The new ICD version 11 therefore should be adjusted to the needs of monitoring of pesticide poisonings.

## Zusammenfassung

**Zielsetzung:** Untersuchung der Häufigkeit, zeitlichen Entwicklung und soziodemographischen Charakteristika der Krankenhausbehandlungen von Pestizidvergiftungen in Deutschland.

**Methoden:** Daten der Diagnosestatistik der aus dem Krankenhaus entlassenen vollstationären Patientinnen und Patienten wurden bezüglich der durch ICD-Schlüssel identifizierbaren Pestizidvergiftungen ausgewertet. Die Häufigkeit, Raten pro eine Million Einwohner und der Anteil Todesfälle wurden jahresbezogen dargestellt. Die Häufigkeit tödlicher Vergiftungen in der Diagnosestatistik wurde der aus der Mortalitätsstatistik gegenüber gestellt.

**Ergebnisse:** Im Zeitraum 2000–2014 wurden 2.817 Pestizidvergiftungen entsprechend durchschnittlich 191 Fällen pro Jahr in deutschen Krankenhäusern behandelt. Die Rate pro 1 Million Einwohner sank von 2,74 im Jahr 2000 auf 1,38 im Jahr 2014. Im Mittel verliefen 5% der Pestizidvergiftungen tödlich, auch der Anteil der tödlichen Vergiftungen halbierte sich im Beobachtungszeitraum. In der Mehrzahl waren Männer von den Vergiftungen betroffen. Bei beiden Geschlechtern kommen ca. 70% der Pestizidvergiftungen bei unter 55-Jährigen vor, ein Drittel betrifft Personen jünger als 25 Jahre. Bei den tödlichen Pestizidvergiftungen ereignen sich indes mehr als 70% der Fälle bei Personen älter als 55 Jahre. Die Krankenhausentlassungsstatistik und Mortalitätsstatistik stimmen nicht gut überein. Im Mittel weist die Diagnosestatistik nur 24% der tödlichen Pestizidvergiftungen der Mortalitätsstatistik aus.

**Schlussfolgerung:** Die Ergebnisse zeigen, dass die Abnahme von Pestizidvergiftungen in Deutschland gleichermaßen bei tödlichen und nicht-tödlichen Fällen beobachtet werden kann. Die sich andeutende Abnahme der Letalität könnte auf positive präventive Effekte einer verringerten Zugänglichkeit von toxischen Pestiziden hindeuten. Tödliche Pestizidvergiftungen kommen vorwiegend bei älteren Männern vor, während die nicht-tödlichen Vergiftungen vermehrt jüngere Personen betreffen. Diese Unterschiede sollten bei der Erarbeitung von Strategien zur Reduktion von Vergiftungen beachtet werden. Die Unterschiede zwischen den beiden betrachteten Statistiken könnten sich durch tödliche Vergiftungsfälle ergeben, die nicht zur Krankenhausaufnahme gelangen. Allerdings ist die hohe Differenz erklärungsbedürftig und sollte weiter untersucht werden. Die ICD erlaubt in der gegenwärtigen Version keine Identifikation von pestiziden Wirkstoffen oder Wirkstoffgruppen. Die derzeit in der Erarbeitung befindliche Version 11 sollte entsprechend angepasst werden, um ein sachgerechtes Monitoring von Pestizidvergiftungen zu ermöglichen.

## Introduction

Human poisoning by pesticides is long since considered a severe public health problem. Although the issue is prevailing and addressed in recent policy papers of international institutions [1], [2], the still most cited figure on acute poisoning was published by the World Health Organization (WHO) as early as 1990 [3]. The taskforce estimated that about one million unintentional pesticide poisonings occur annually leading to approximately 20,000 deaths worldwide. Authors pointed to a vast underreporting as most incidents occur in countries with no effective monitoring of poisonings. More recent figures are available for self-poisoning by pesticides which in a systematic review is estimated to amount to 260,000–370,000 deaths per year mostly occurring in rural agricultural areas in low- and middle-income countries [4]. The authors also point to the persistent lack of good quality data even in countries with high public health standards which hampers the monitoring of country specific trends in pesticide poisonings and the evaluation of the effects of preventive strategies.

In Germany, pesticide poisoning has lately been studied based on the cause-of-death statistics [5]. In a 30-year period fatal pesticides poisonings in Germany declined by about 90% reaching 39 deaths in the year 2010. As a possible reason for this impressive decline – which is not visible in all countries – a limited access to substances especially of high toxicity has been discussed. However, if this were the case the reduction of pesticide poisonings would not apply to non-fatal incidents in general. 

Estimating non-fatal pesticide poisoning is challenging. In Germany an obligation for physicians to report on acute poisonings was introduced by law (§16 Chemikaliengesetz) in 1990. However, numbers from this reporting scheme still differ considerably from other statistics. E.g. none of the aforementioned 39 deaths due to pesticide poisoning in the year 2010 were reported to the health authorities [6]. Complete coverage of hospital discharged cases is provided by the so-called statistics of diagnoses (Diagnosestatistik) which is obligatory facilitated by all German hospitals. This database will still lead to an underestimation of the occurrence of poisonings as on the one hand milder poisonings might not lead to hospital treatment. On the other hand, poisonings might end fatal before cases are recognized and admitted to hospitals. In Germany, the ambulant treatment by general practitioners builds the main branch in medical care. However, we expect that poisonings are transferred to hospitals rather generally as surgeries do not provide monitoring due to limited opening hours. Furthermore, all German poison control centers are located at hospitals. Consequently, with respect to non-fatal pesticide poisonings we expect that most cases will be captured by the diagnoses statistics. Incalculable underreporting however can occur from undetected poisonings especially in case of chronic poisoning. 

The aim of this study was to analyze the frequency, trends, and patterns of hospital treated pesticide poisoning in Germany. We will focus on non-fatal cases but additionally study the agreement of numbers of fatal pesticide poisoning in the hospital discharge statistics and the official mortality statistics.

## Methods

We analyzed data form the German statistics of diagnoses of hospital discharges which is provided annually by the German Statistical Office [7]. The data comprise information on hospital discharges from all hospitals in Germany. Reporting is obligatory for every hospital and covers the main diagnosis at time of discharge as well as information of the patients (sex, age) and of the case (duration, county). The “main” diagnosis refers to the disease which is – after final examinations – considered the cause of hospital admission. The diagnosis is coded by the 4-digit International Classification of Diseases (ICD) Version 10. Fatal cases are differentiated but reported by a 3-digit ICD code only. ICD codes related to pesticides are provided in Table 1 (Tab. 1).

In order to study the number and trends of pesticides poisonings we analyzed data from 2000 to 2014. Prior the year 2000 reporting was based on ICD version 9 which provided a different coding scheme for pesticides poisonings and comparability is limited. We present year-specific crude numbers of cases for specific ICD codes. For pesticide poisoning in total (ICD T60) we additionally calculated rates per one million inhabitants by dividing the crude number of cases by the annual population of Germany [8]. We give the number of fatal cases along with the percentage of all pesticides related discharges. Finally, we compared the number of fatal pesticides related poisonings to the official German causes-of-death statistics by compiling respective ICD codes and years [5], [9].

## Results

Table 2 (Tab. 2) gives the annual numbers of hospital treated pesticide poisonings in Germany. In the 15 years studied 2,871 pesticides poisonings were treated amounting to 191 cases per year on the average. Overall the annual number of cases decreased and the rate per 1 million almost halved from 2.74 in 2000 to 1.38 in 2014. On average 5% of pesticides poisonings were fatal. This percentage also approximately halved from 2000 to 2014. 

With respect to the pesticide groups organophosphates and carbamate insecticides (T60.0) had the greatest overall share causing 29% of all pesticide poisonings. However, from 2008 on most causes are referred to as “other pesticides” (T60.8). Overall in 44% of incidents pesticides were not specified or belong to “other” categories (T60.2, T60.8, T60.9).

The majority of pesticide poisonings occurred in men whose share increased from 57% in 2000 to 64% in 2014. In both sexes approximately 70% of all pesticide poisonings occurred at an age of less than 55 years and one third in patient younger than 25 (Table 3 (Tab. 3)). This is different with respect to fatal poisonings with men sharing almost 80% and more than 70% of cases occurring above 55 years of age. No fatal pesticide poisonings affected women younger than 25 years.

**Figure 1:**
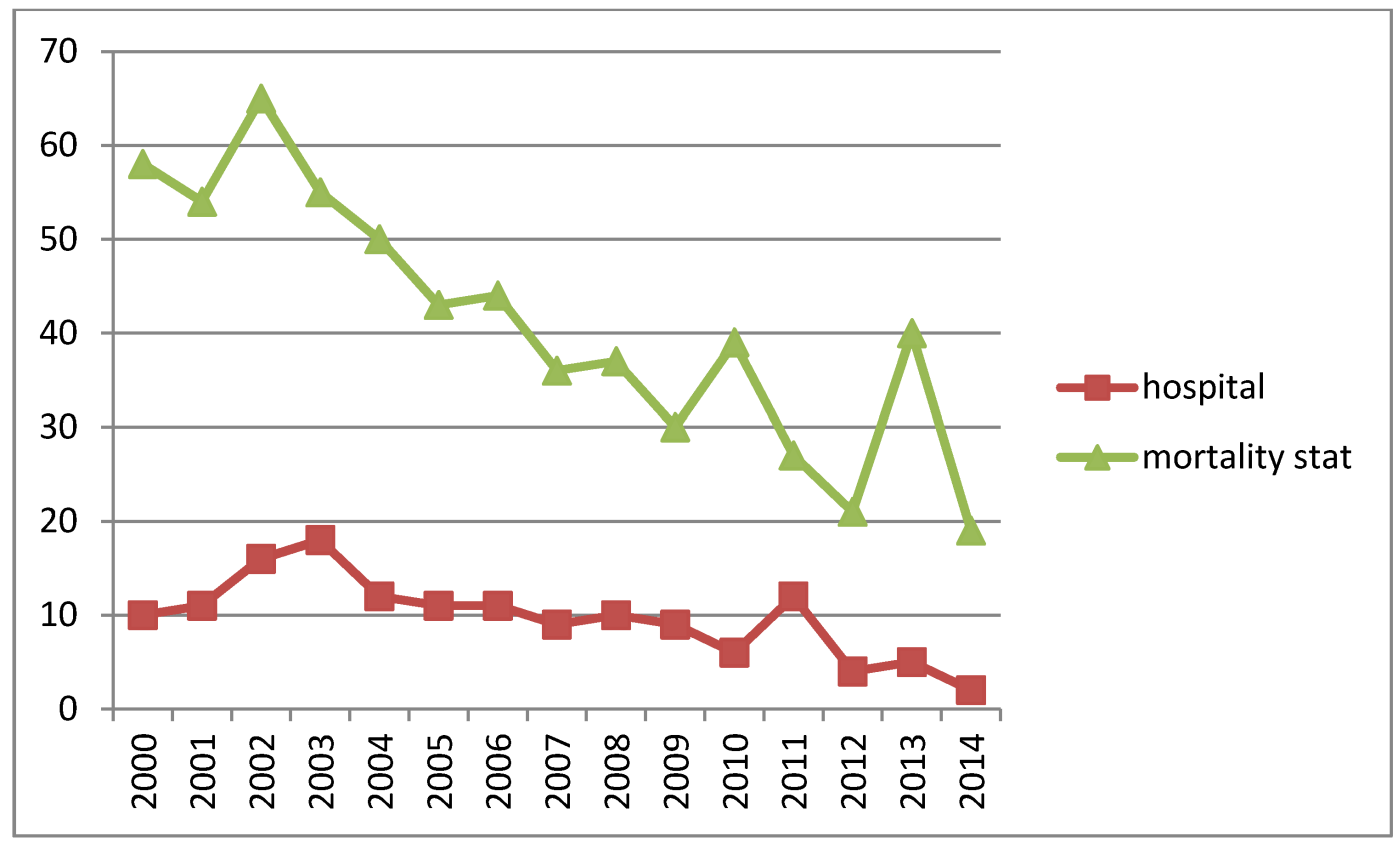
Comparison of numbers of fatal pesticide poisonings in diagnoses statistics (hospital) and mortality statistics (mortality stat). Source: see references [7], [5], [9]

The comparison of the number of fatal cases from the diagnoses statistics to the official mortality statistics shows considerable differences. On the average only 24% of the fatalities in the mortality statistics were shown in the diagnosis statistics ranging from 44% in 2011 to 11% in 2014 with no clear pattern (Figure 1 (Fig. 1)).

## Discussion and conclusion

This study shows that the decrease of pesticide poisonings in Germany is not restricted to the fatal incidents. However, the reduction is found to be more prominent in fatal pesticide poisonings and there seems to be a decrease also in the percentage of fatal cases. Fatal pesticide poisonings are mainly due to suicides and the reduced availability of toxic pesticides is considered a main cause for the decline [2], [5]. Still, the situation might be different for non-intentional poisoning. Safe use strategies additional to worker and consumer protection laws which restrict the access to pesticides especially for untrained users might have been effective. Unfortunately, the hospital discharge statistics does not distinguish suicidal from accidental poisoning. We observed differences in the sex and age distribution of pesticides poisonings with respect to the outcomes. Fatal pesticides poisonings which are known to follow predominantly from suicidal intention prevails in elder men while non-fatal pesticide poisoning relatively often affects the younger population.

This study shows a mismatch between the statistics with respect to fatal pesticide poisoning. E.g., in 2014 the number of fatalities shown in the hospital discharge statistics is only approximately one tenth of that in the mortality statistics. There may be two reasons for this discrepancy. First, intoxicated persons might not be found in time and die prior admittance to a hospital. This could be a probable scenario particularly in case of intentional poisonings. Second, the different coding regime leads to misrepresentation. The mortality statistics is based on death certificates and therefore the coded diagnosis is the underlying disease (for death). In contrast, the hospital discharge statistics retrieves the cause of admission as so called main diagnosis. E.g. a fatal pesticide poisoning following a depressive episode might get coded as depressive disorder in the death certificate and as pesticide poisoning in hospital discharge. However, in this scenario an underreporting in the mortality statistics can be expected what is in contrast to our results. 

The suitability of the ICD for monitoring pesticide poisonings is limited. In order to support preventive strategies an identification of the active ingredients or at least the chemical group of a pesticide should be captured. The ICD version 10 does not serve this purpose as most incidents refer to not specified pesticides or ones that belong to “other” categories. Obviously, the ICD coding system does not capture the changed market of pesticides, so that codes like T60.1 for halogenated insecticides find hardly a counterpart in the real world. Currently, the new ICD version 11 is underway. Unfortunately, the beta draft version shows no improvement so far [10]. 

Our paper has some limitations. As this is a descriptive study of existing register data we rely on the validity of the retrieved information. The diagnoses statistics is considered to represent the complete number of hospital treated cases. The quality of the ICD codes however depends on the compliance of hospital staff and cannot be validated from outside. Finally, register data do not provide information to analyze influencing and confounding factors of observed trends. We refrained from calculating age standardized rates because of small number of age and ICD code-specific cases.

## Notes

### Competing interests

The authors declare that they have no competing interests.

## Figures and Tables

**Table 1:**
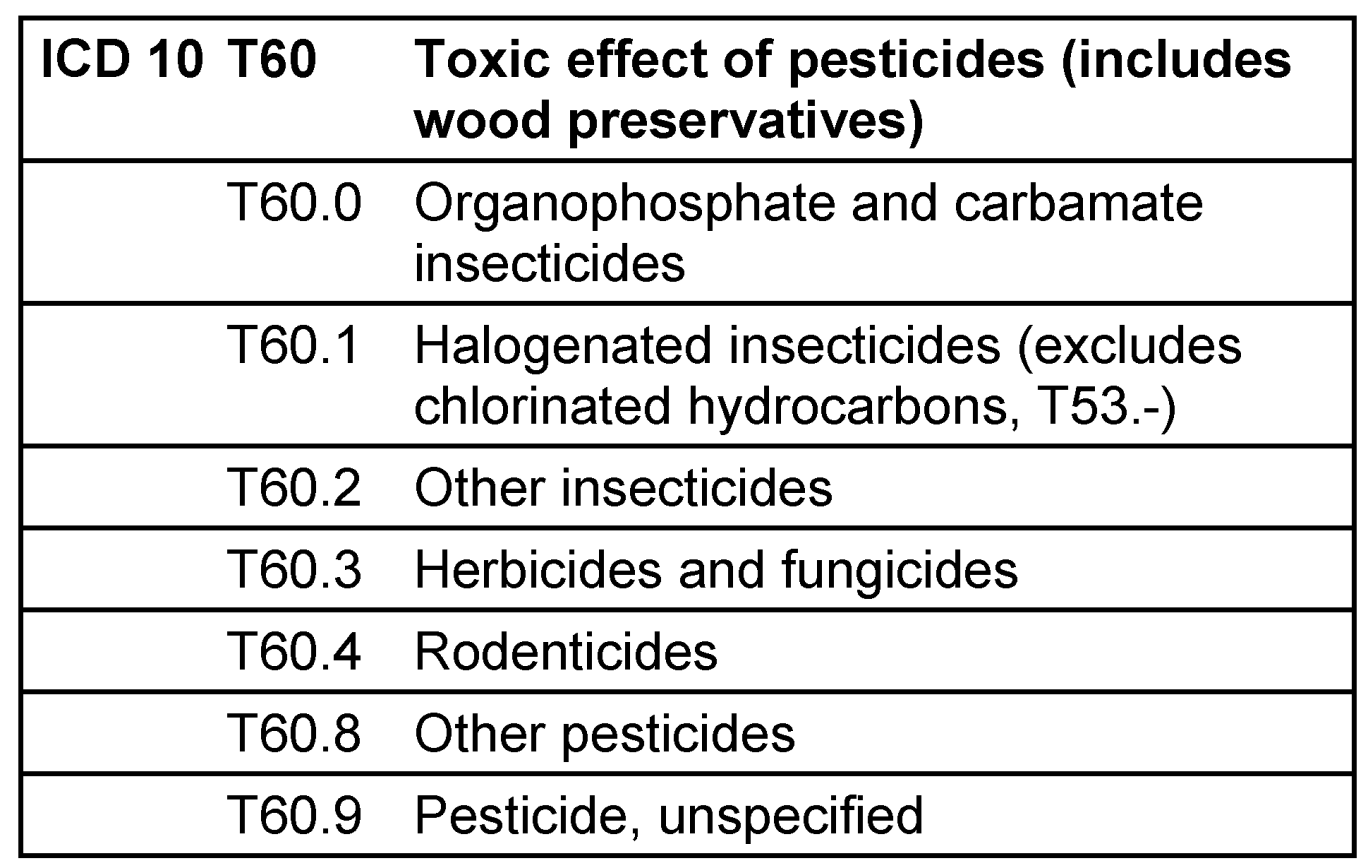
International Classification of Disease codes (ICD 10) referring to pesticide poisonings

**Table 2:**
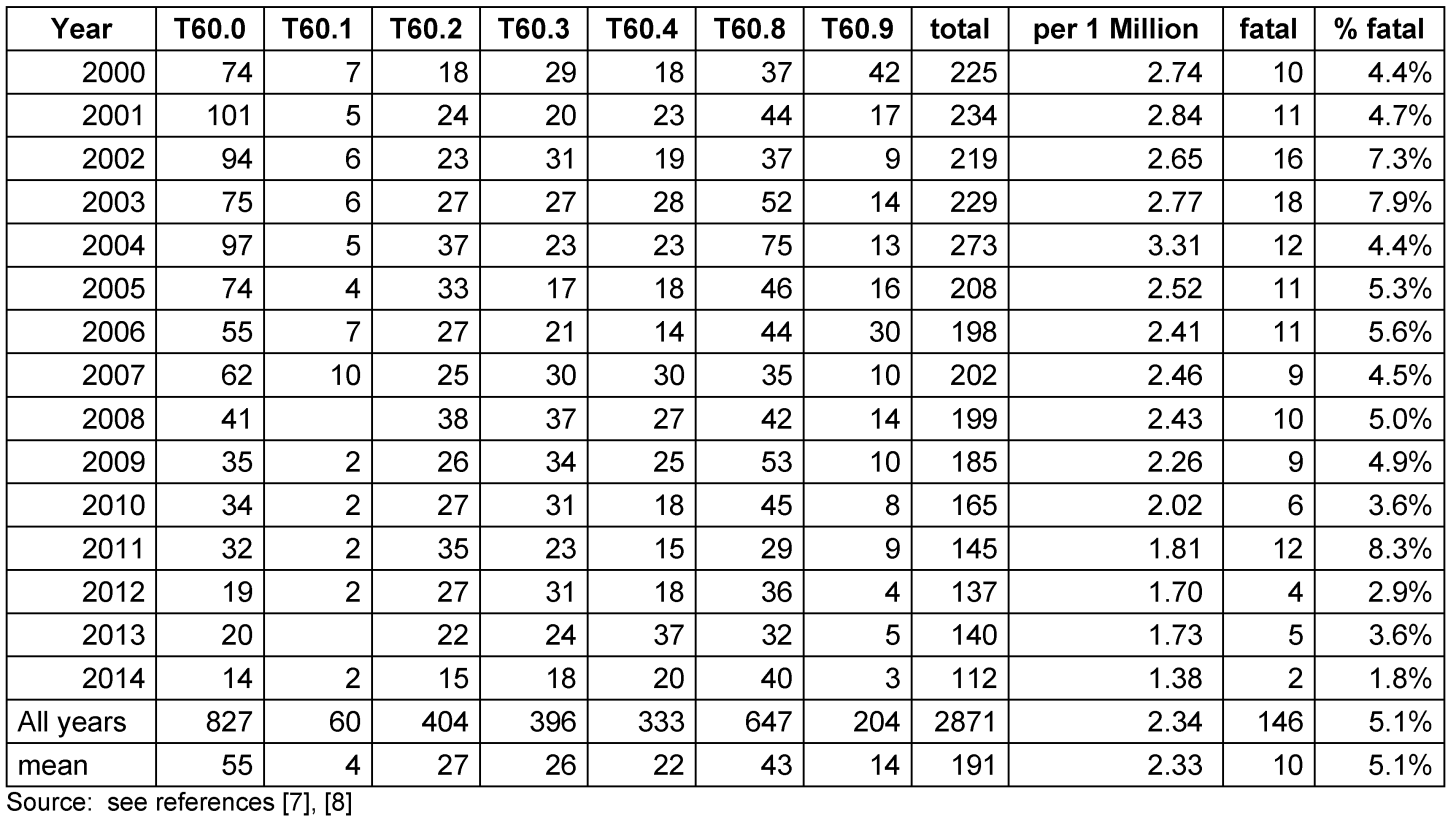
Number and rates of hospital treated pesticide poisonings in Germany 2000–2014 according to ICD10 codes.

**Table 3:**
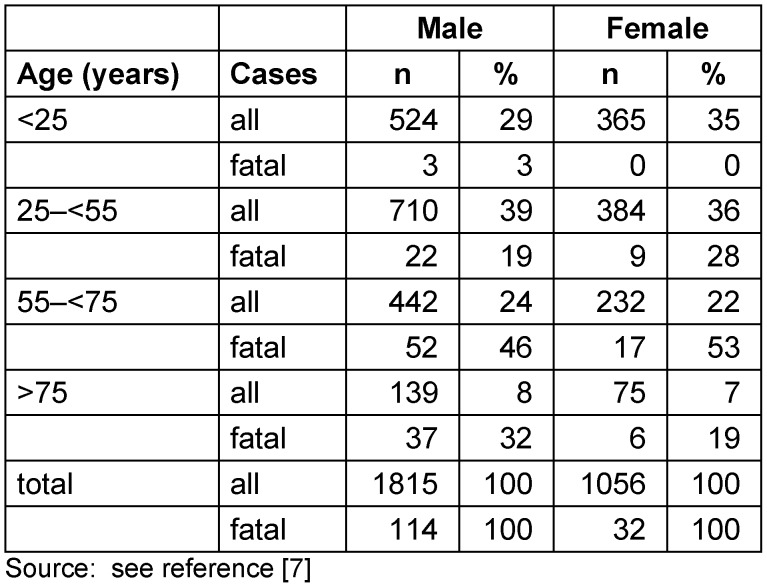
Hospital treated pesticide poisonings in Germany 2000–2014 by sex and age
